# Supplementary material for: Enhanced Bactericidal Effect of Calcinated Mg–Fe Layered Double Hydroxide Films Driven by the Fenton Reaction
Source: Int J Mol Sci. 2022 Dec 23;24(1):272. doi: 10.3390/ijms24010272 (PMC9820372; doi:10.3390/ijms24010272)
Supplement: Supplementary file 1 [file ijms-24-00272-s001.zip › ijms-2058439-supplementary.pdf]

International Journal of *Molecular Sciences*

MDPI

Supporting Information

Enhanced Bactericidal Effect of Calcinated Mg–Fe Layered Double Hydroxide Films Driven by the Fenton Reaction

Lei Chen [1,†], Yijia Yin [2,†], Linjia Jian [1], Xianglong Han [2], Xuefeng Zhao [2,*] and Donghui Wang [3,*]

1 School of Materials Science and Engineering, Hebei University of Technology, Tianjin 300130, China
2 State Key Laboratory of Oral Diseases & National Clinical Research Center for Oral Diseases & Department of Orthodontics, West China Hospital of Stomatology, Sichuan University, Chengdu 610041, China
3 School of Health Sciences and Biomedical Engineering, Hebei University of Technology, Tianjin 300130, China
* Correspondence: zhao.axf@gmial.com (X.Z.); donghuiwang@hebut.edu.cn (D.W.)
† These authors contributed equally to this work.

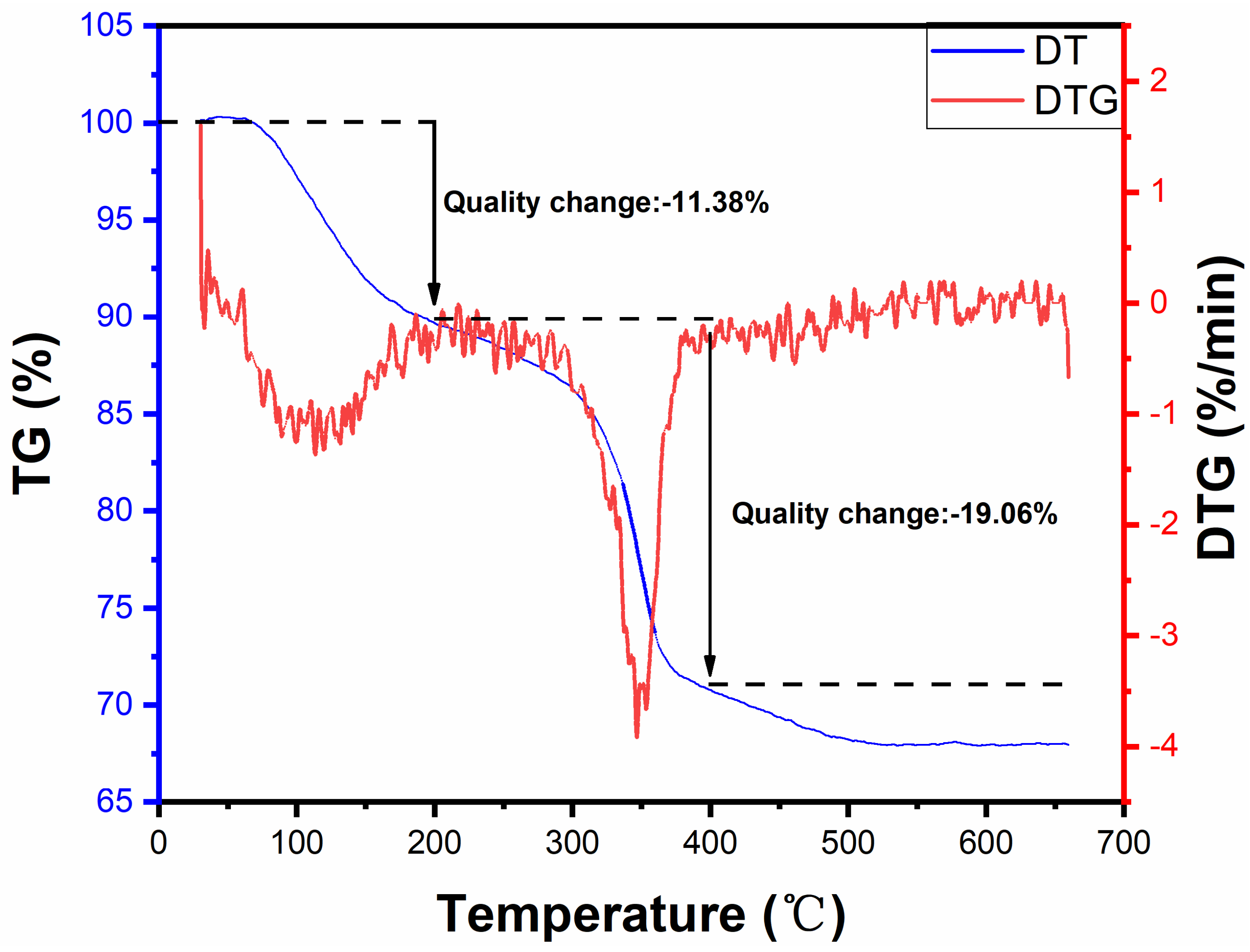

Figure S1. Thermogravimetric analysis of LDH−1 samples.

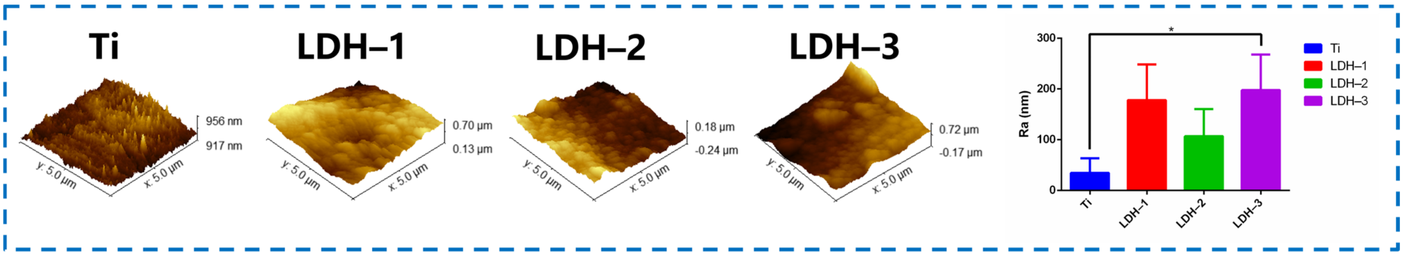

Figure S2. The roughness of Ti, LDH−1, LDH−2 and LDH−3 sample surfaces. (* $p < 0.05$).

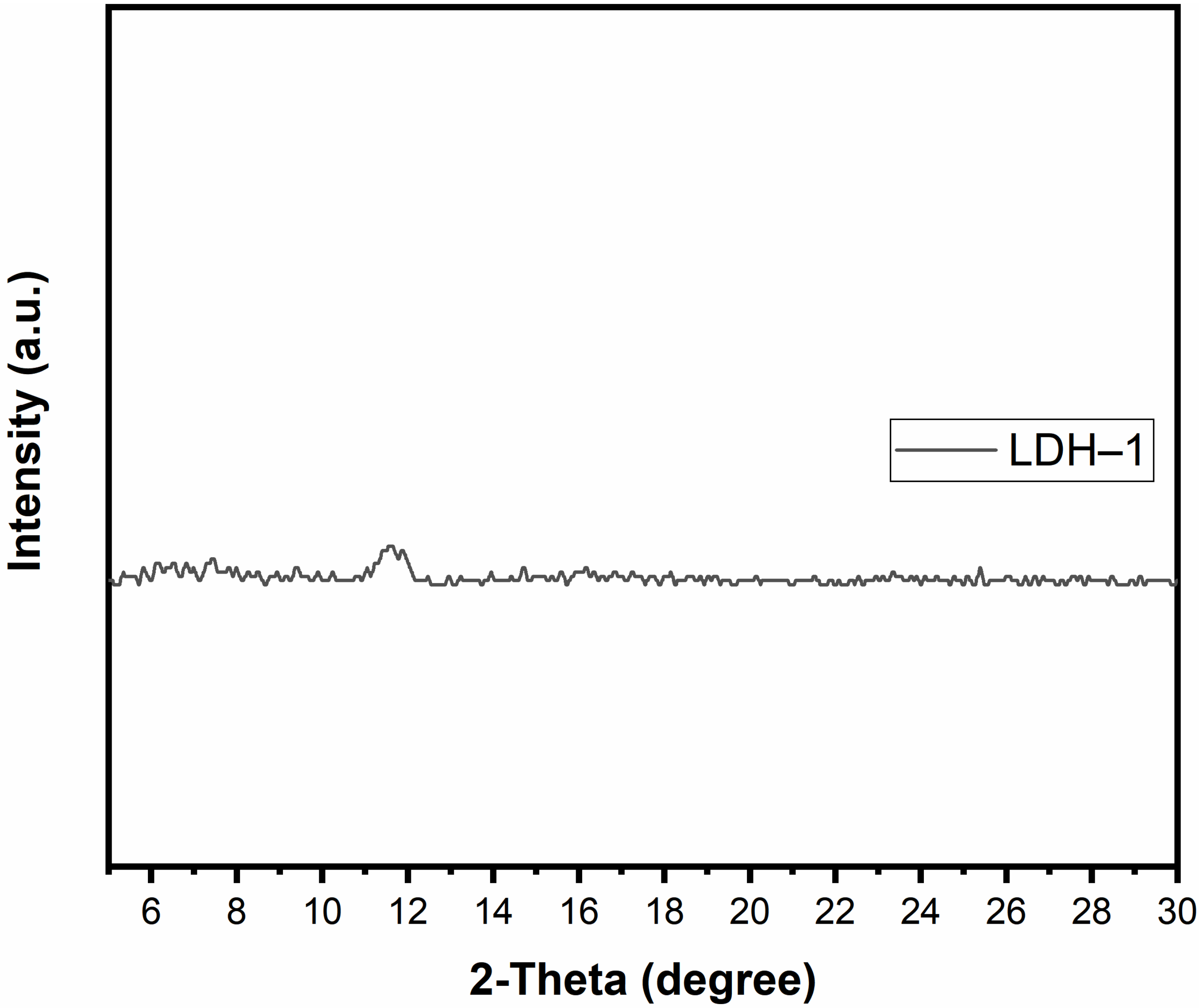

Figure S3. The XRD pattern of LDH−1 sample.

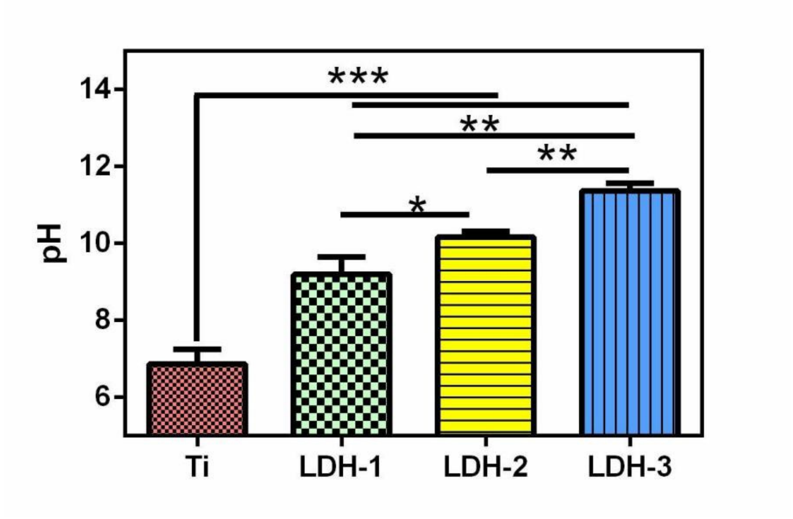

Figure S4. Local alkaline microenvironment of different samples [1]. (*$p < 0.05$, **$p < 0.01$, ***$p < 0.001$).

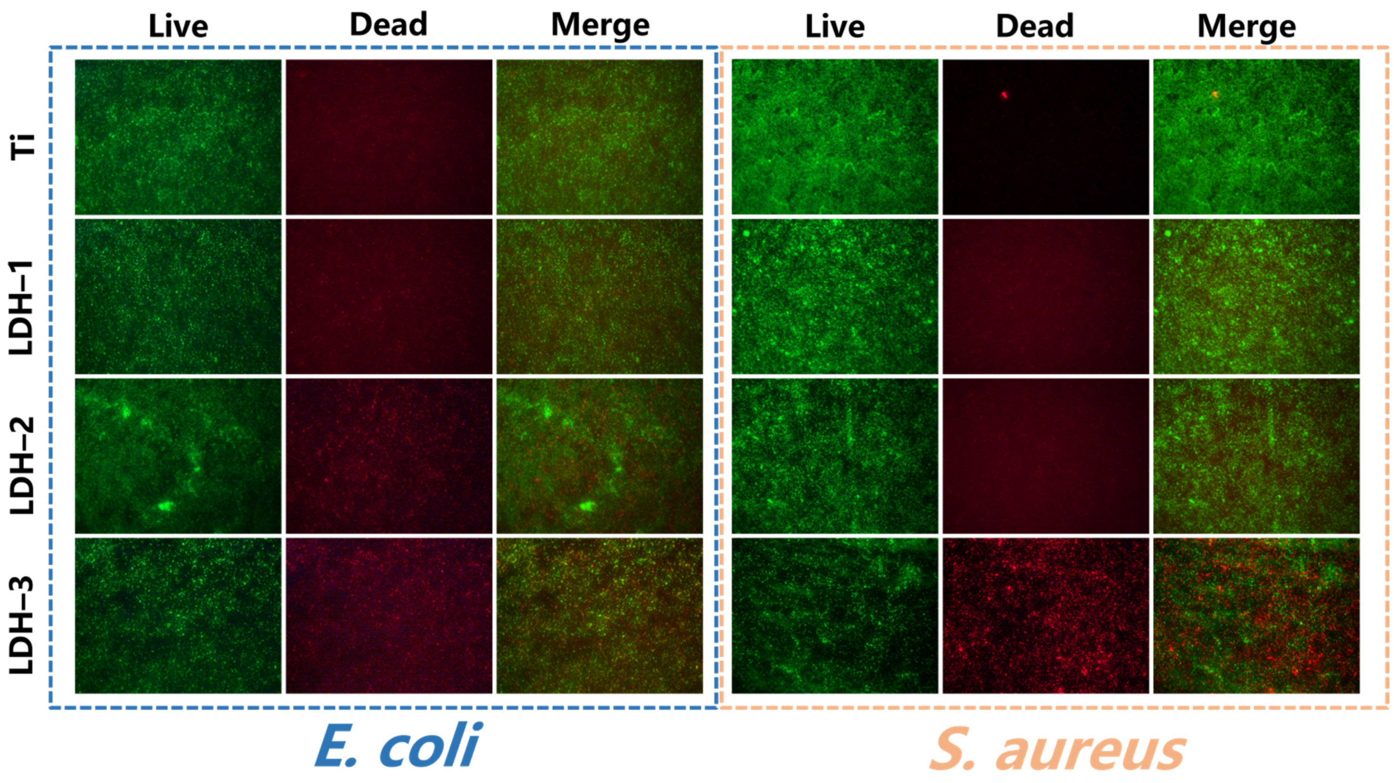

Figure S5. The fluorescent images of the live (green)/ dead (red) staining of E. coli and S. aureus cultured on Ti, LDH–1, LDH–2 and LDH–3 sample surfaces for 1 day.

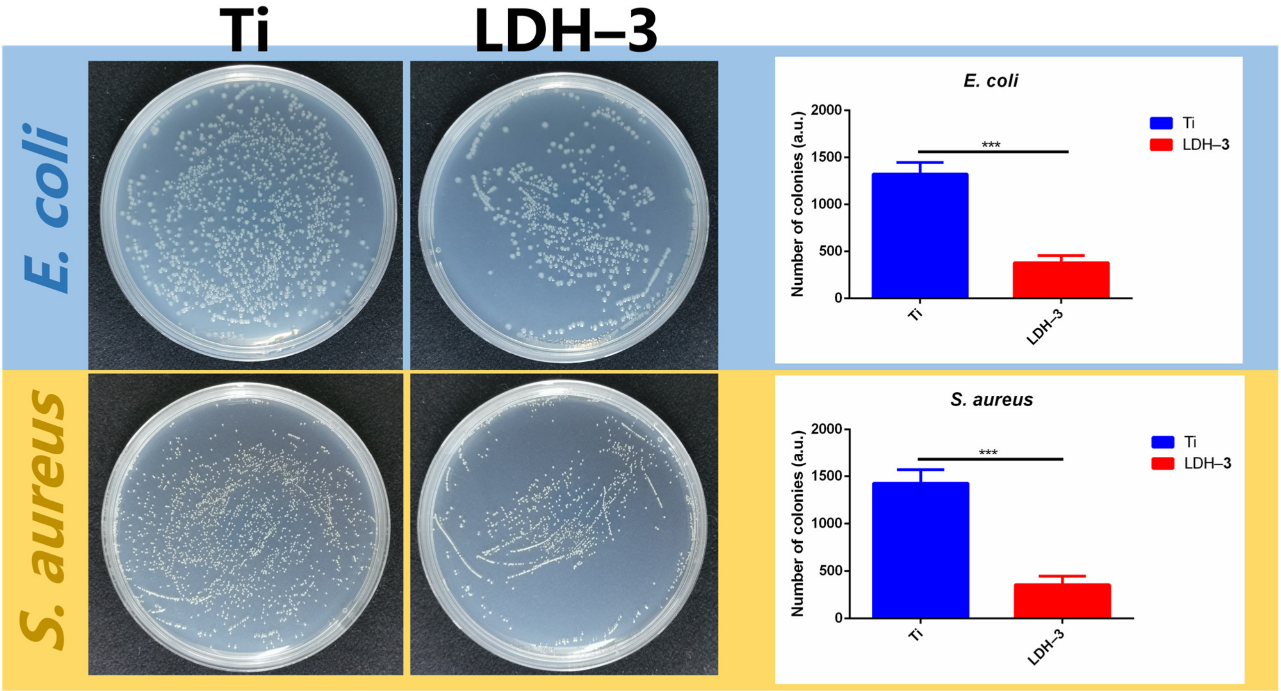

Figure S6. The photographs of colonies distribution of S. aureus and E. coli cultured on Ti and LDH−3 samples under an acidic microenvironment containing 5 μM H_2O_2 and corresponding quantitative analysis. (***$p < 0.001$).

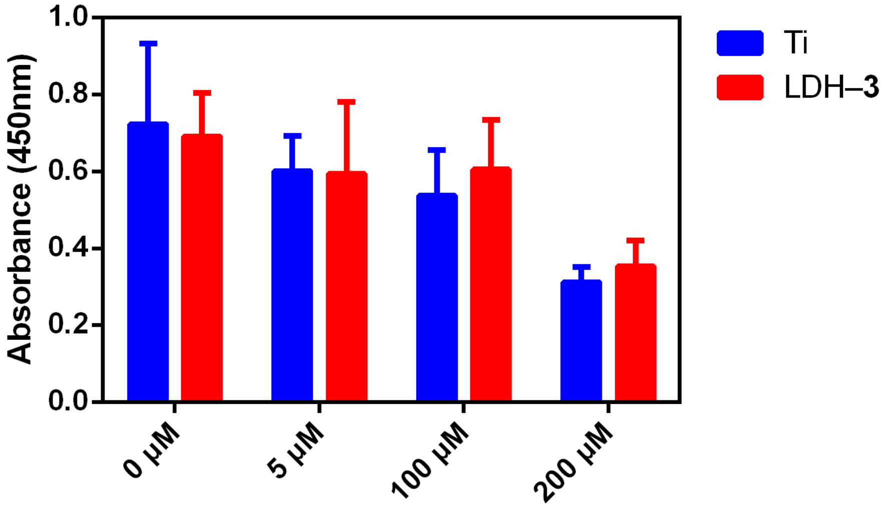

Figure S7. BMMSCs proliferation activity on Ti, and LDH−3 sample surfaces under 0, 5, 100, and 200 μM H_2O_2.

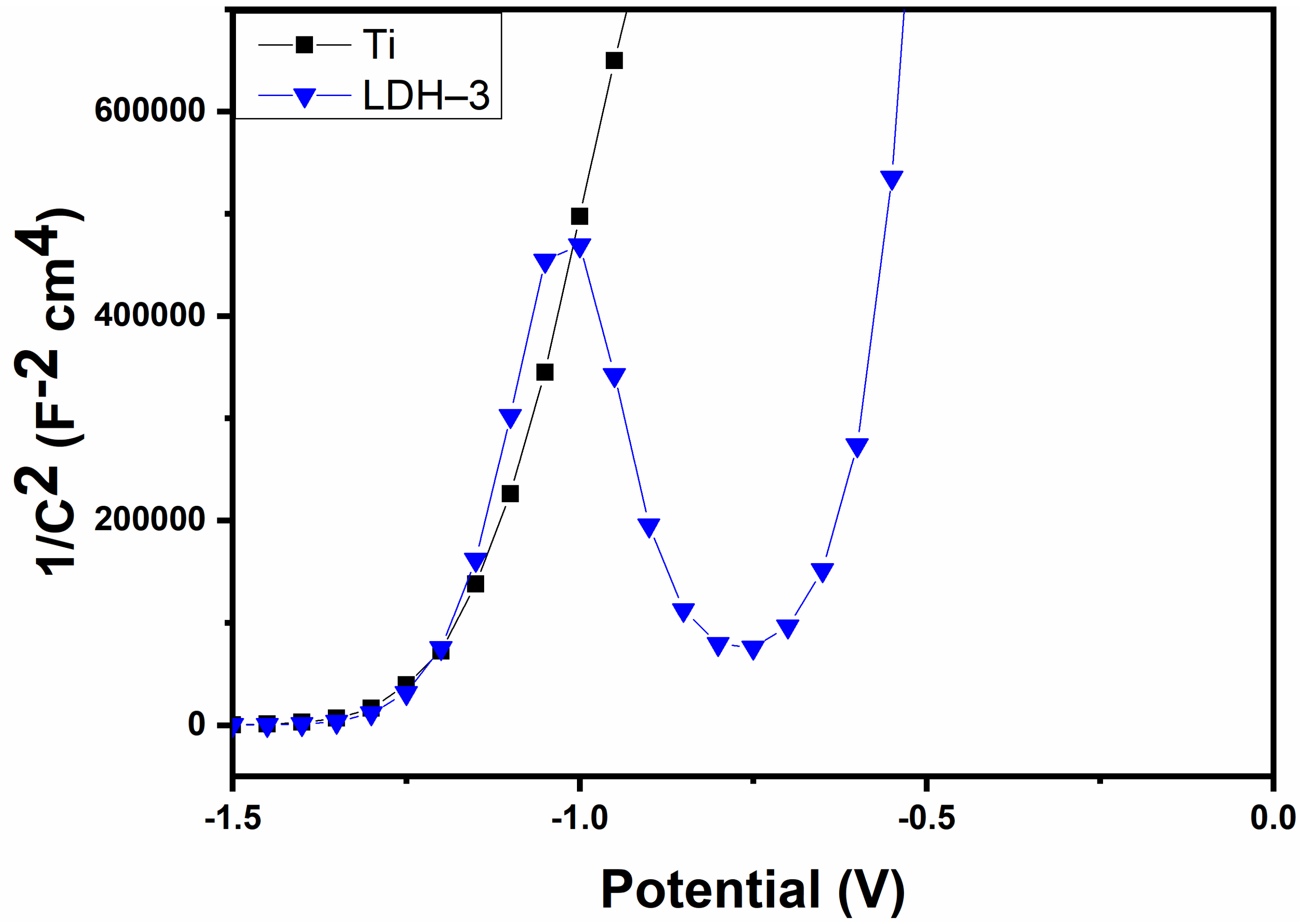

Figure S8. Mott–Schottky curves of Ti and LDH–3 samples.

Table S1. The carrier concentration of different samples calculated from the MS curves.

	Ti	LDH–3
Ne	9.42E19	7.06E19
Nh	/	7.06E12

1. Yin, Y.; Jian, L.; Li, B.; Liang, C.; Han, X.; Zhao, X.; Wang, D., Mg-Fe layered double hydroxides modified titanium enhanced the adhesion of human gingival fibroblasts through regulation of local pH level. *Mater. Sci. Eng. C Mater. Biol. Appl.* **2021,** *131*, 112485.
